# Supplementary material for: Lactobacillus rhamnosus colonisation antagonizes Candida albicans by forcing metabolic adaptations that compromise pathogenicity
Source: Nat Commun. 2022 Jun 9;13:3192. doi: 10.1038/s41467-022-30661-5 (PMC9184479; doi:10.1038/s41467-022-30661-5)
Supplement: Supplementary file 1 — Supplementary Information [file 41467_2022_30661_MOESM1_ESM.pdf]

Supplementary material for

# *Lactobacillus rhamnosus* colonisation antagonizes *Candida albicans* by forcing metabolic adaptations that compromise pathogenicity

Raquel Alonso-Roman[1], Antonia Last[1], Mohammad H. Mirhakkak[2], Jakob L. Sprague[1], Lars Möller[1], Peter Großmann[2], Katja Graf[1,3], Rena Gratz[1], Selene Mogavero[1], Slavena Vylkova[4], Gianni Panagiotou[2,5], Sascha Schäuble[2], Bernhard Hube[1,6], Mark S. Gresnigt[7]

**Corresponding author**: bernhard.hube@hki-jena.de

**Affiliations:**

1: Department of Microbial Pathogenicity Mechanisms, Leibniz Institute for Natural Product Research and Infection Biology - Hans-Knoell-Institute, Jena, Germany

2: Systems Biology and Bioinformatics Unit, Leibniz Institute for Natural Product Research and Infection Biology - Hans-Knoell-Institute, Jena, Germany

3: Dynamic42 GmbH, Jena, Germany

4: Septomics Research Centre, Leibniz Institute for Natural Product Research and Infection Biology – Hans-Knoell-Institute, Jena, Germany

5: Department of Medicine and State Key Laboratory of Pharmaceutical Biotechnology, University of Hong Kong, China

6: Institute of Microbiology, Friedrich Schiller University, Jena, Germany

7: Junior Research Group Adaptive Pathogenicity Strategies, Leibniz Institute for Natural Product Research and Infection Biology - Hans-Knoell-Institute, Jena, Germany

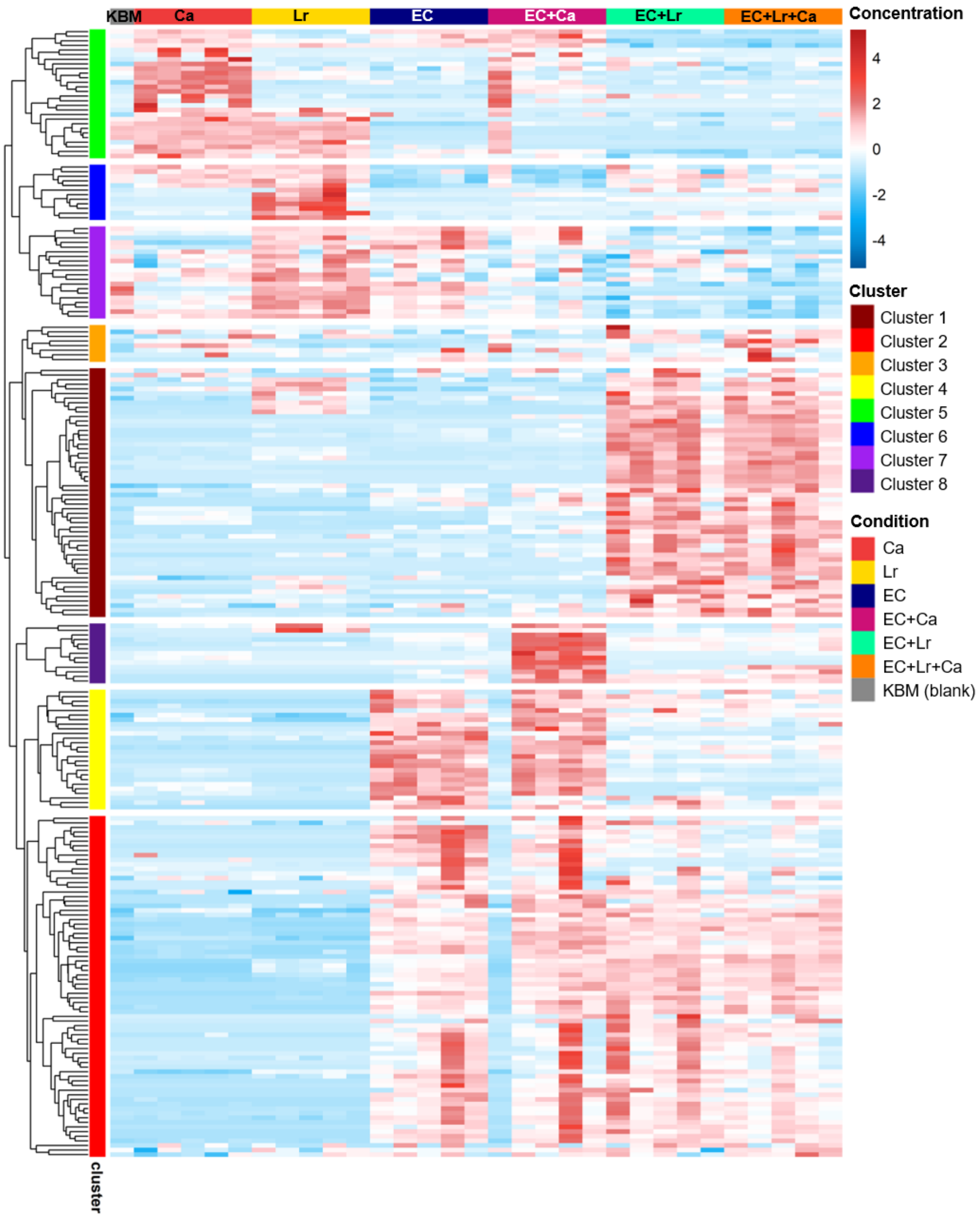


**Supplementary Fig. 1: Untargeted metabolomics of *in vitro* intestinal epithelial *L. rhamnosus* colonization and *C. albicans* infection**

Hierarchical clustering of the presence and absence of metabolites in the *in vitro* model at 12 hours post infection (hpi).

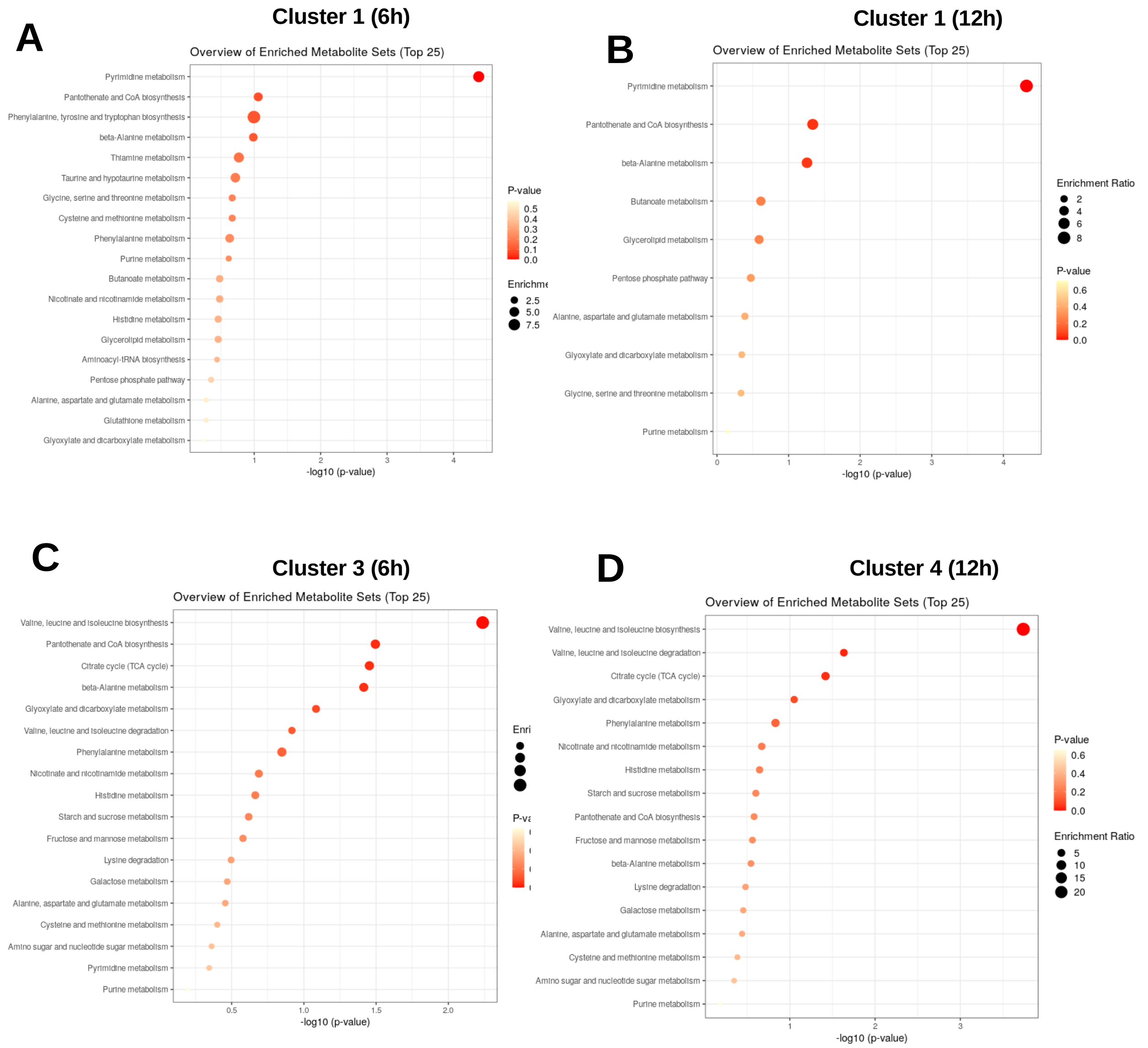


**Supplementary Fig. 2: Enriched metabolic pathways**

Overview of the top 25 enriched metabolic sets in the supernatants analysed by a hypergeometric test in the clusters of (A) metabolites produced by IECs and *L. rhamnosus* in synergy at 6h and (B) 12h; and (C) produced by IECs and consumed by *L. rhamnosus* at 6h and (D) 12h. Dot size represents enrichment ratio obtained with overrepresentation analysis and colour of the dots represents *p*-value (FDR-correction applied, FDR ≤ 0.1 was considered).

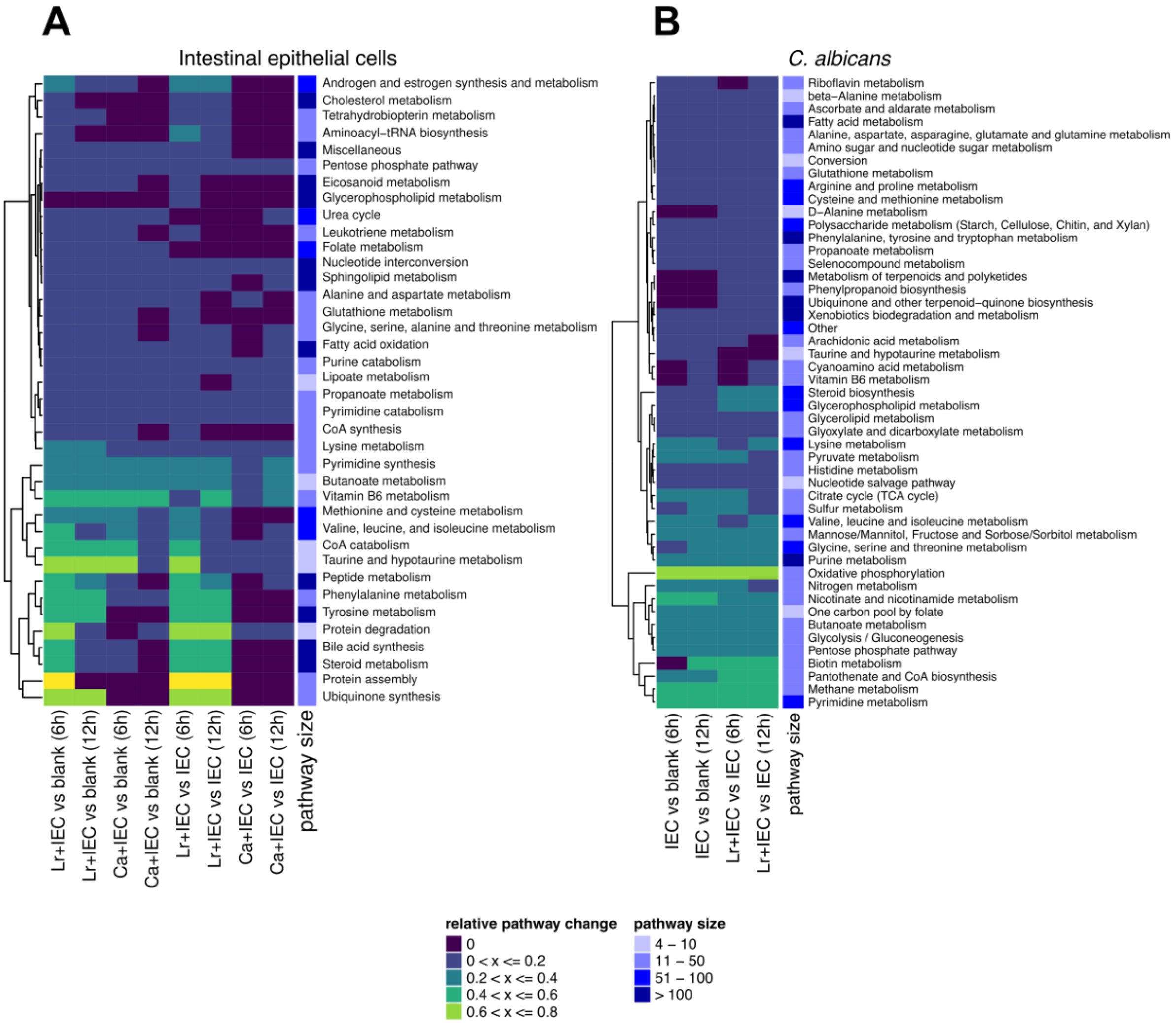


**Supplementary Fig. 3: Pathway flux activity of IECs and *C. albicans***

Simulation of pathway activity levels in IECs (A) and in *C. albicans* (B) compared between different setups as indicated. Relative pathway change was determined by identifying the number of pathway-specific reactions for which feasible flux ranges differ according to flux variability analysis.

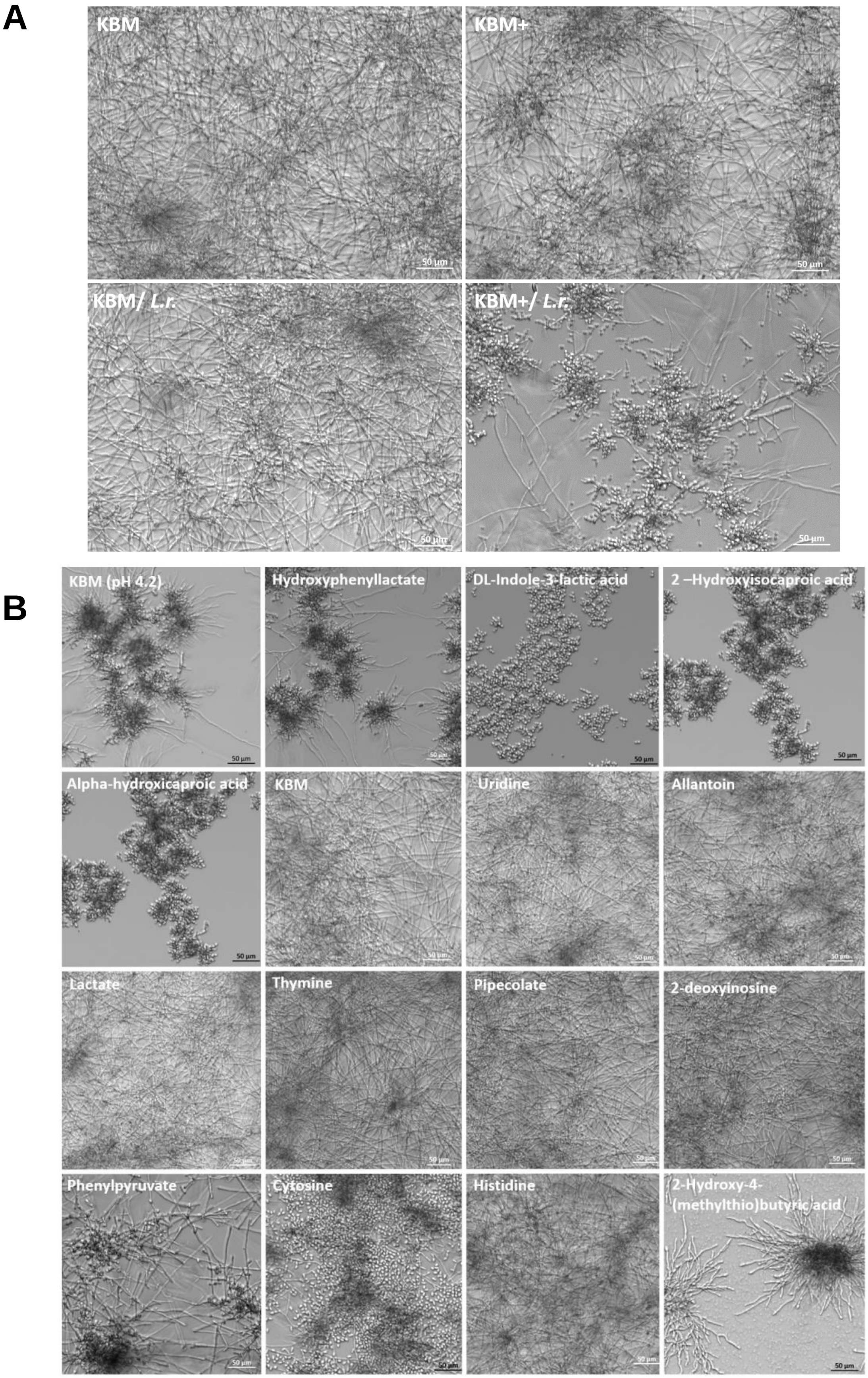
A
KBM
KBM+
KBM/ L.r.
KBM+/ L.r.
50 µm
B
KBM (pH 4.2)
Hydroxyphenyllactate
DL-Indole-3-lactic acid
2 –Hydroxyisocaproic acid
Alpha-hydroxicaproic acid
KBM
Uridine
Allantoin
Lactate
Thymine
Pipecolate
2-deoxyinosine
Phenylpyruvate
Cytosine
Histidine
2-Hydroxy-4-(methylthio)butyric acid

**Supplementary Fig. 4: Impact of *L. rhamnosus*-colonization derived metabolites on *C. albicans***

(A) Representative images of *C. albicans* morphology changes grown in *L. rhamnosus*-conditioned or unconditioned media, after 20 h at 37°C with 5% $CO_2$ (n=3 biological replicates). (B) Representative images of *C. albicans* morphology grown in presence of all the metabolites tested, at neutral or acidic pH, after 20 h at 37°C with 5% $CO_2$ (n=2 biological replicates).

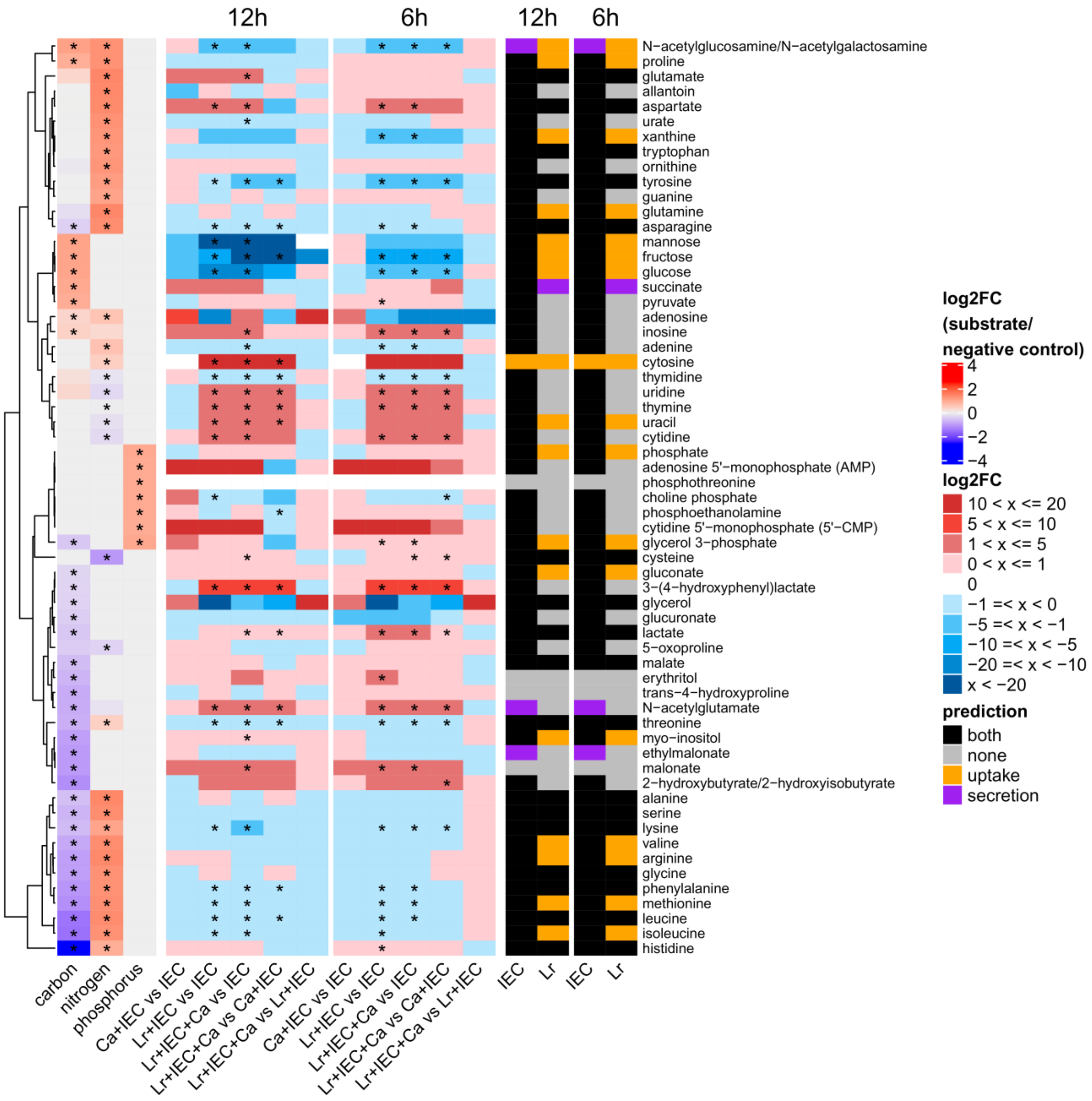


**Supplementary Fig. 5: *L. rhamnosus* colonization creates an unfavourable metabolic environment for *C. albicans***

Phenotypic microarray growth experiments for wild-type *C. albicans* on a carbon, nitrogen or phosphorous source (left). Metabolome data measured at 6 and 12 h as well as metabolic modelling predictions (right) are indicated for the whole panel of metabolites. For metabolic modelling, media was adapted from metabolome data derived from IECs spent media or blank. Uptake or secretion was determined by identifying feasible flux ranges for metabolite-specific exchange reactions alongside optimization for biomass. ANOVA (two-sided) was performed for phenotypic microarrays, Wilcoxon test (two-sided) for metabolomics, with FDR correction (* = $p_{adj} \leq 0.05$).

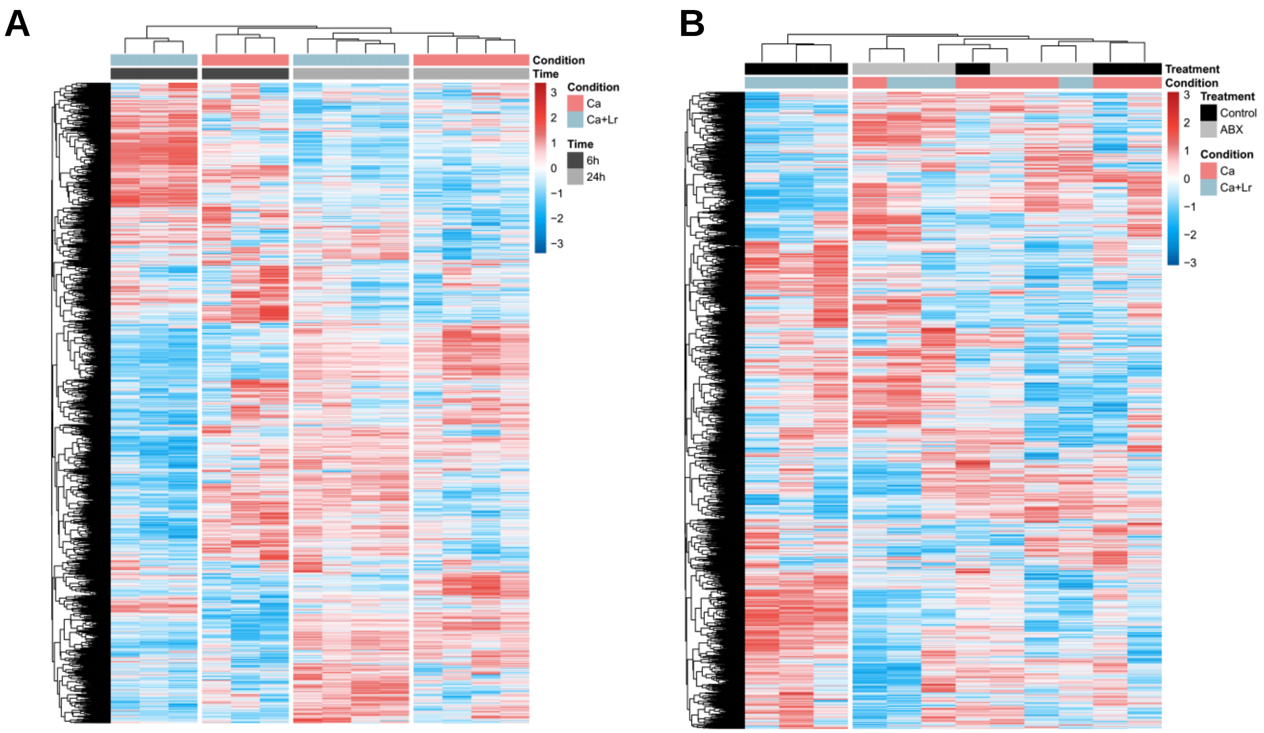


**Supplementary Fig. 6: Changes in *C. albicans* gene expression upon infection of *L. rhamnosus* colonized IECs**

(A) Hierarchical clustering based on Euclidean distance of *C. albicans* gene expression at 6 and 24 h during *in vitro* infection of IECs in the presence and absence of *L. rhamnosus* colonization. Data summarized from n=3 and n=4 independent experiments at 6 and 24 hpi respectively. (B) Hierarchical clustering based on Euclidean distance of *C. albicans* gene expression during *in vitro* infection of IECs in the presence and absence of *L. rhamnosus* colonization and in the presence and absence of antibiotics. Data summarized from n=3 at 24 hpi.

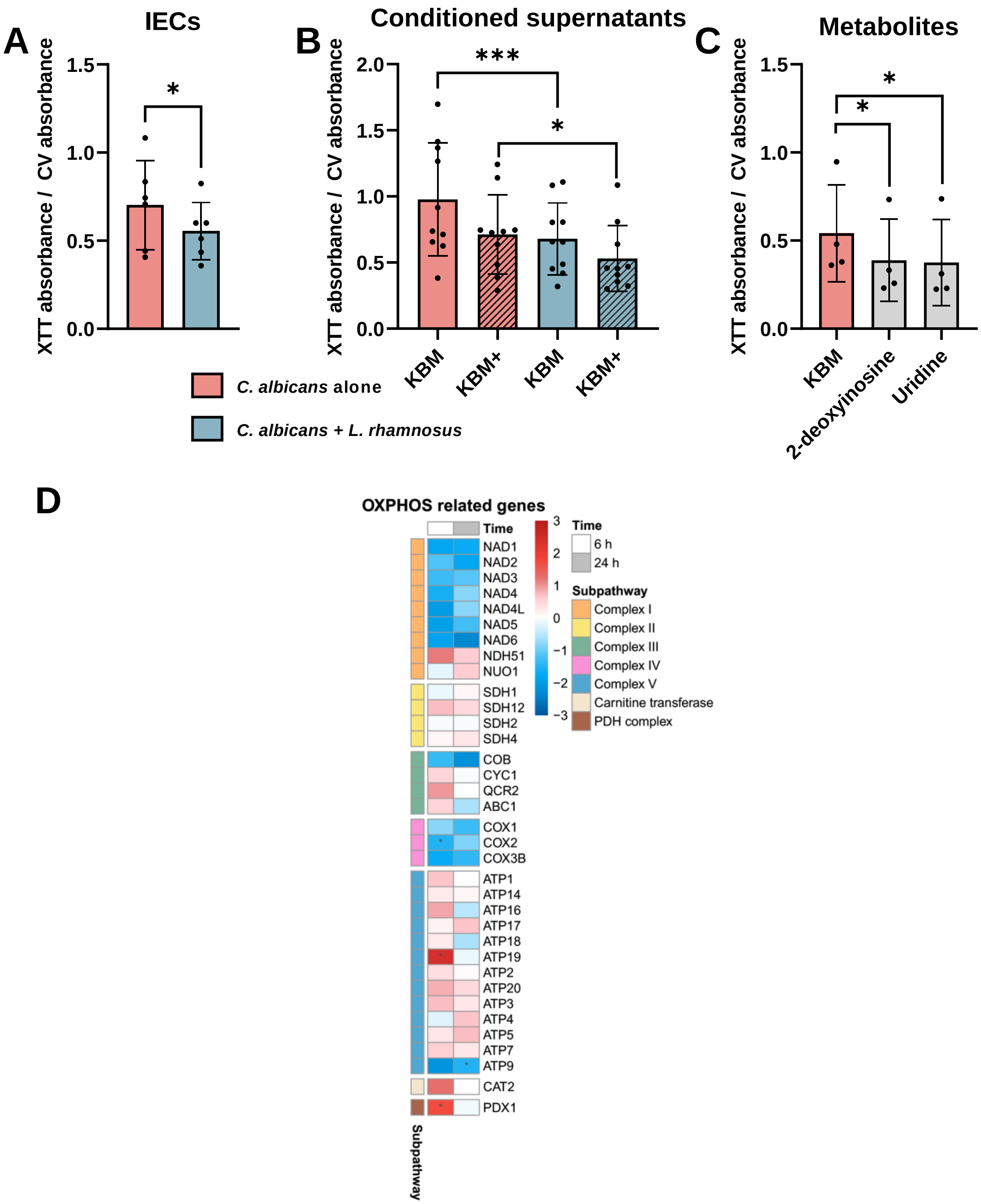


**Fig. S7 *L. rhamnosus* modulates activity of *C. albicans* mitochondrial dehydrogenases**

(A) Mitochondrial activity, represented as conversion of XTT by mitochondrial dehydrogenases (XTT absorbance) normalized with biomass (crystal violet (CV) absorbance, of *C. albicans* incubated in supernatants of *L. rhamnosus*-colonized or uncolonized IECS (1h, 37°C, 5% $CO_2$) (n=6, * = *p* 0.0350), or (B) in *L. rhamnosus*-conditioned or unconditioned KBM or KBM+ medium independently of host cells (1h, 37°C, 5% $CO_2$) (n=10, *** = *p* 0.0007 for KBM and * = *p* 0.0170 for KBM+), or (C) in presence or absence of 50 mM uridine (* = *p* 0.0470 )or 2-deoxyinosine (* = *p* 0.0278) (24h, 37°C, 5% $CO_2$) (n=4). Bars represent the mean and error bars show standard

deviation (SD), dots represent the mean of technical replicates of the individual experiments, data were compared for significance using a paired t-test (two-tailed), * = $p \leq 0.05$, ** = $p \leq 0.01$, *** = $p \leq 0.001$. Source data are provided as a Source Data file. (D) Heatmap showing the transcriptional regulation of *C. albicans* OXPHOS genes as a result of *L. rhamnosus* colonization prior to infection at 6 and 24 hpi. Legend colour represents the $Log_2$ fold change of the regulation in presence *vs.* absence of *L. rhamnosus*. The asterisks (*) represent significance, based on the criteria of a $Log_2$ fold change of > 1 or < -1 and a moderated t-test Bonferroni corrected *p*-value of < 0.05. Data summarized from n=3 and n=4 independent experiments at 6 and 24 hpi respectively.

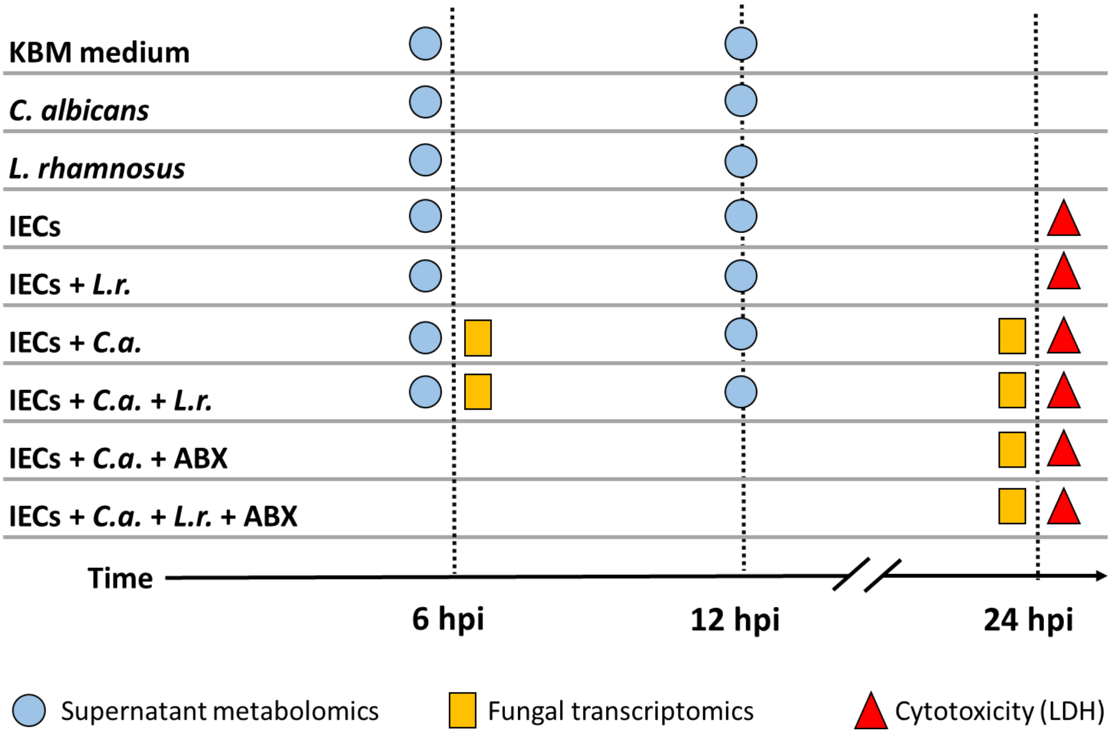


**Supplementary Fig. 8: Study design**

Overview of the samples, taken at different time points for transcriptional, metabolic, and cytotoxicity analysis in the *in vitro* model, in the presence of intestinal epithelial cells (IECs), *L. rhamnosus* (*L.r.*), *C. albicans* (*C.a.*) and antibiotics (ABX).

## Supplementary Table 1: Metabolites of the clusters 1-8 shown in Fig. 2B (6 hpi).

| **Cluster 1**<br>“Secreted by *L. rhamnosus* colonized IECs” |
|---|
| (N(1) + N(8))-acetylspermidine |
| 1-carboxyethyltyrosine |
| 1-palmitoyl-2-linoleoyl-GPC (16:0/18:2) |
| 2'-deoxycytidine |
| 2'-deoxyinosine |
| 2-hydroxy-3-methylvalerate |
| 2-hydroxy-4-(methylthio)butanoic acid |
| 2-hydroxybutyrate/2-hydroxyisobutyrate |
| 2-hydroxyglutarate |
| 2R,3R-dihydroxybutyrate |
| 3-(4-hydroxyphenyl)lactate |
| 3-formylindole |
| 3-hydroxyoctanoate |
| 3-indoleglyoxylic acid |
| 4-methylthio-2-oxobutanoate |
| 5-methyluridine (ribothymidine) |
| allantoin |
| alpha-hydroxyisocaproate |
| alpha-hydroxyisovalerate |
| benzoate |
| cysteine |
| cytidine |
| cytosine |
| dihydroorotate |
| glycerate |
| glycerophosphoglycerol |
| histidine |
| hypoxanthine |
| imidazole lactate |
| indolelactate |
| lactate |
| N-acetylglutamate |
| N-acetylproline |
| N-acetylthreonine |
| N-acetyltryptophan |
| N-acetyltyrosine |
| N-carbamoylaspartate |
| N-formylmethionine |
| nicotinamide riboside |
| nicotinate ribonucleoside |
| orotate |
| phenyllactate (PLA) |
| phenylpyruvate |
| phosphate |
| pipecolate |
| p-toluic acid |
| sedoheptulose |
| thioproline |
| thymine |
| trimethylamine N-oxide |
| uracil |
| uridine |

| **Cluster 2**<br>“Secreted by IECs” |
|---|
| 1-carboxyethylisoleucine |
| 1-carboxyethylleucine |
| 1-carboxyethylphenylalanine |
| 1-carboxyethylvaline |
| 1-methylnicotinamide |
| 1-palmitoyl-2-oleoyl-GPC (16:0/18:1) |
| 1-stearoyl-2-oleoyl-GPC (18:0/18:1) |
| 2'-deoxycytidine |
| 2'-deoxyuridine |
| 2'-O-methylcytidine |
| 3-hydroxy-3-methylglutarate |
| 3-hydroxybutyrate (BHBA) |

| **Cluster 2**<br>“Secreted by IECs”<br>Continued |
|---|
| 3-hydroxyisobutyrate |
| 4-acetamidobutanoate |
| 4-imidazoleacetate |
| 7-methylguanine |
| alanine |
| Allantoin |
| alpha-ketoglutarate |
| 4-guanidinobutanoate |
| arabonate/xylonate |
| argininosuccinate |
| Benzoate |
| beta-alanine |
| Betaine |
| cholesterol |
| choline phosphate |
| creatine |
| creatinine |
| cystathionine |
| dimethylarginine (SDMA + ADMA) |
| erythritol |
| erythronate |
| ethylmalonate |
| fumarate |
| gamma-glutamyl-alpha-lysine |
| gamma-glutamylthreonine |
| glucuronate |
| glycerol 3-phosphate |
| glycerophosphoethanolamine |
| glycerophosphoglycerol |
| glycerophosphoinositol |
| glycerophosphorylcholine (GPC) |
| glycerophosphoserine |
| glycine |
| guanine |
| gulonate |
| homoarginine |
| hypotaurine |
| hypoxanthine |
| kynurenine |
| Lactate |
| malate |
| mannitol/sorbitol |
| methionine sulfone |
| N1-methyladenosine |
| N1-methylinosine |
| N6,N6,N6-trimethyllysine |
| N-acetylalanine |
| N-acetyl-isoputreanine |
| N-acetylleucine |
| N-acetylneuraminate |
| nicotinamide ribonucleotide (NMN) |
| nicotinamide riboside |
| N-monomethylarginine |
| ornithine |
| palmitoyl sphingomyelin (d18:1/16:0) |
| pantothenate |
| p-cresol sulfate |
| phenol red |
| phenol sulfate |
| phenylacetylglycine |
| proline |
| pseudouridine |
| pyridoxal |
| pyridoxamine |
| pyridoxate |
| pyridoxine (Vitamin B6) |
| ribonate |

| **Cluster 2**<br>“Secreted by IECs”<br>Continued |
|---|
| succinate |
| sulfate |
| taurine |
| thiamin (Vitamin B1) |
| threonate |
| trans-4-hydroxyproline |
| Urate |

| **Cluster 3**<br>“Secreted by IECs consumed by *L. rhamnosus”* |
|---|
| 1-ribosyl-imidazoleacetate |
| 2'-O-methyluridine |
| 3-methyl-2-oxobutyrate |
| 3-ureidopropionate |
| 4-methyl-2-oxopentanoate |
| 5-methylthioadenosine (MTA) |
| acetylcarnitine (C2) |
| aconitate [cis or trans] |
| carnitine |
| carnosine |
| citrate |
| cysteinylglycine disulfide |
| deoxycarnitine |
| fructose |
| gamma-glutamylalanine |
| gamma-glutamylglutamine |
| gamma-glutamylhistidine |
| gamma-glutamylisoleucine |
| gamma-glutamylleucine |
| gamma-glutamylmethionine |
| gamma-glutamylserine |
| Hippurate |
| Isocitrate |
| N-acetylglucosamine/N-acetylgalactosamine |
| N-acetylmethionine |
| nicotinamide |
| S-1-pyrroline-5-carboxylate |
| Xanthine |

| **Cluster 4**<br>“Consumed by IECs” |
|---|
| 2'-deoxyadenosine |
| 3-sulfo-L-alanine |
| Adenosine |
| alpha-lipoate |
| Arginine |
| Aspartate |
| cysteine s-sulfate |
| cysteine sulfinic acid |
| Cystine |
| Gluconate |
| Glutamate |
| Glutamine |
| Inosine |
| Isoleucine |
| Leucine |
| mannonate |
| methionine |
| methionine sulfoxide |
| N-acetylarginine |
| N-acetylglutamine |
| phenylalanylhydroxyproline |
| phosphoethanolamine |
| phosphothreonine |
| Serine |
| Valine |

| **Cluster 5**<br>“Secreted by *C. albicans*” |
|---|
| 2,3-dihydroxyisovalerate |
| 4-hydroxyphenylpyruvate |
| folate |
| glutarate (C5-DC) |
| glycerol |
| kynurenate |

| **Cluster 6**<br>“Inconclusive” |
|---|
| 2-oxoarginine |
| 3-hydroxybutyroylglycine |
| 5-oxoproline |
| adenosine 5'-monophosphate (AMP) |
| biotin |
| choline |
| cytidine 5'-monophosphate (5'-CMP) |
| myo-inositol |
| N-acetylasparagine |
| N-acetylaspartate (NAA) |
| N-acetylhistidine |
| pyroglutamine |
| tryptophan |

| **cluster 7**<br>“Consumed by *L. rhamnosus* colonized IECs” |
|---|
| adenine |
| arabitol/xylitol |
| asparagine |
| cysteine-glutathione disulfide |
| fructosyllysine |
| gamma-glutamylvaline |
| Glucose |
| Lysine |
| phenylalanine |
| Ribitol |
| riboflavin (Vitamin B2) |
| threonine |
| thymidine |
| Tyrosine |

| **Cluster 8**<br>“Secreted by *L. rhamnosus* alone” |
|---|
| alpha-ketoglutaramate |
| argininate |
| diacetylspermidine |
| glutamine conjugate of C6H10O2 (2) |
| malonate |
| methylsuccinate |
| mevalonate |
| mevalonolactone |
| N-acetylleucine |
| N-acetylisoleucine |
| N-acetylphenylalanine |
| N-acetylputrescine |
| N-acetylserine |
| pyruvate |

## Supplementary Table 2: Metabolites of the clusters 1-8 shown in Fig. S1 (12 hpi).

| Cluster 1<br>“Secreted by *L. rhamnosus* colonized IECs” |
|---|
| (N(1) + N(8))-acetylspermidine |
| 1-carboxyethyltyrosine |
| 2'-deoxyinosine |
| 2-hydroxy-3-methylvalerate |
| 2-hydroxy-4-(methylthio)butanoic acid |
| 2-hydroxybutyrate/2-hydroxyisobutyrate |
| 2-hydroxyglutarate |
| 2R,3R-dihydroxybutyrate |
| 3-(4-hydroxyphenyl)lactate |
| 3-formylindole |
| 3-hydroxyoctanoate |
| 3-indoleglyoxylic acid |
| 3-ureidopropionate |
| 5-methyluridine (ribothymidine) |
| alpha-hydroxyisocaproate |
| alpha-hydroxyisovalerate |
| cytidine |
| cytosine |
| dihydroorotate |
| glycerate |
| indolelactate |
| mevalonate |
| mevalonolactone |
| N-acetylglutamate |
| N-acetylthreonine |
| N-acetyltryptophan |
| N-acetyltyrosine |
| N-carbamoylaspartate |
| nicotinate ribonucleoside |
| phenyllactate (PLA) |
| pipecolate |
| sedoheptulose |
| thymine |
| uracil |
| uridine |

| Cluster 2<br>“Secreted by IECs” |
|---|
| 1-carboxyethylisoleucine |
| 1-carboxyethylleucine |
| 1-carboxyethylphenylalanine |
| 1-carboxyethylvaline |
| 1-methylnicotinamide |
| 1-palmitoyl-2-oleoyl-GPC (16:0/18:1) |
| 1-stearoyl-2-oleoyl-GPC (18:0/18:1) |
| 2'-deoxycytidine |
| 2'-deoxyuridine |
| 2'-O-methylcytidine |
| 3-hydroxy-3-methylglutarate |
| 3-hydroxybutyrate (BHBA) |
| 3-hydroxyisobutyrate |
| 4-imidazoleacetate |
| 7-methylguanine |
| Alanine |
| Allantoin |
| alpha-ketoglutarate |
| arabonate/xylonate |
| argininosuccinate |
| Benzoate |
| beta-alanine |
| betaine |

| Cluster 2<br>“Secreted by IECs” continued |
|---|
| cholesterol |
| choline phosphate |
| creatine |
| creatinine |
| cystathionine |
| dimethylarginine (SDMA + ADMA) |
| erythritol |
| erythronate |
| ethylmalonate |
| fumarate |
| gamma-glutamylthreonine |
| glucuronate |
| glycerol 3-phosphate |
| glycerophosphoethanolamine |
| Glycerophosphoglycerol |
| Glycerophosphoinositol |
| glycerophosphorylcholine (GPC) |
| Glycerophosphoserine |
| Gulonate |
| Homoarginine |
| Hypotaurine |
| Hypoxanthine |
| Lactate |
| malate |
| mannitol/sorbitol |
| methionine sulfone |
| N1-methyladenosine |
| N1-methylinosine |
| N6,N6,N6-trimethyllysine |
| N-acetylalanine |
| N-acetyl-isoputreanine |
| N-acetylleucine |
| N-acetylneuraminate |
| nicotinamide riboside |
| N-monomethylarginine |
| ornithine |
| Palmitoyl sphingomyelin (d18:1/16:0) |
| pantothenate |
| p-cresol sulfate |
| phenol sulfate |
| pseudouridine |
| pyridoxal |
| pyridoxamine |
| pyridoxate |
| ribonate |
| Sulfate |
| taurine |
| trans-4-hydroxyproline |
| Urate |

| Cluster 3<br>“Inconclusive” |
|---|
| 1-palmitoyl-2-linoleoyl-GPC (16:0/18:2) |
| 3-hydroxybutyroylglycine |
| benzoate |
| cholesterol |
| imidazole lactate |
| methylsuccinate |
| N-acetylaspartate (NAA) |
| N-acetylproline |

| Cluster 4<br>“Secreted by IECs and consumed by *L. rhamnosus*” |
|---|
| 1-ribosyl-imidazoleacetate |
| 3-methyl-2-oxobutyrate |
| 3-methyl-2-oxovalerate |
| 4-methyl-2-oxopentanoate |
| 5-methylthioadenosine (MTA) |
| acetylcarnitine (C2) |
| aconitate [cis or trans] |
| carnitine |
| carnosine |
| citrate |
| deoxycarnitine |
| fructose |
| gamma-glutamylalanine |
| gamma-glutamylglutamine |
| gamma-glutamylhistidine |
| gamma-glutamylisoleucine |
| gamma-glutamylleucine |
| gamma-glutamylmethionine |
| gamma-glutamylserine |
| gamma-glutamylvaline |
| hippurate |
| isocitrate |
| N-acetylglucosamine/N-acetylgalactosamine |
| N-acetylmethionine |
| nicotinamide |
| S-1-pyrroline-5-carboxylate |
| xanthine |

| Cluster 5<br>“Secreted by *C. albicans*” |
|---|
| 2,3-dihydroxyisovalerate |
| 2'-deoxyadenosine |
| 3-sulfo-L-alanine |
| 4-methylthio-2-oxobutanoate |
| adenosine |
| alpha-lipoate |
| arabitol/xylitol |
| aspartate |
| benzoate |
| cysteine s-sulfate |
| glycerol |
| imidazole lactate |
| kynurenate |
| N-acetylleucine |
| N-acetylputrescine |
| phenylalanylhydroxyproline |
| ribitol |

| Cluster 6<br>“Secreted by *L. rhamnosus* alone” |
|---|
| adenosine |
| argininate |
| diacetylspermidine |
| gluconate |
| glutamine conjugate of C6H10O2 (2) |
| Inosine |
| malonate |
| N-acetylisoleucine |
| N-acetylphenylalanine |
| N-acetylserine |
| phosphothreonine |

| cluster 7<br>“Consumed by *L. rhamnosus* colonized IECs” |
|---|
| adenine |
| arginine |
| asparagine |
| biotin |
| cystine |
| fructosyllysine |
| glucose |
| glutamine |
| HEPES |
| isoleucine |
| leucine |
| lysine |
| mannitol/sorbitol |
| mannose |
| methionine |
| phenylalanine |
| serine |
| Threonine |
| tryptophan |
| valine |

| Cluster 8<br>“Secreted by *C. albicans* infected IECs” |
|---|
| cysteine-glutathione disulfide |
| indoleacetate |
| isovalerate (i5:0) |
| nicotinamide ribonucleotide (NMN) |

## Supplementary Table 3: Abbreviations used in Fig.6

| Abbreviation | Full name |
| --- | --- |
| ***Reactions*** | |
| ACO | Aconitate Hydratase |
| AKGDH | alpha-Ketoglutarate Dehydrogenase |
| ALPL | 2-Acetolactate Pyruvate-Lyase |
| CAR2 | Ornithine Aminotransferase |
| ccoN | Cytochrome c Oxidase |
| CS | Citrate Synthase |
| DLD | D-lactate Dehydrogenase |
| ENO | Enolase |
| FBA | Fructose-Bisphosphate Aldolase |
| fbcH | Ubiquinol-Cytochrome c Reductase |
| FBP | Fructose-1,6-Bisphosphatase |
| FUM | Fumarate Hydratase |
| G6PD | Glucose-6-phosphate 1-Dehydrogenase |
| GAPDH | Glyceraldehyde 3-Phosphate Dehydrogenase |
| GPI | Glucose-6-Phosphate Isomerase |
| HK | Hexokinase |
| HPPR | Hydroxyphenylpyruvate Reductase |
| IDH | Isocitrate Dehydrogenase |
| ISL | Isocitrate Lyase |
| LDH | L-Lactate Dehydrogenase |
| MDH | Malate Dehydrogenase |
| MS | Malate Synthase |
| nuoA | NADH-Quinone Oxidoreductase |
| PCK | Phosphoenolpyruvate Carboxykinase |
| PDH | Pyruvate Dehydrogenase |
| PFK | 6-Phosphofructokinase |
| PGAM | Phosphoglycerate Mutase |
| PGD | 6-Phosphogluconate Dehydrogenase |
| PGK | Phosphoglycerate Kinase |
| PGL | 6-Phosphogluconolactonase |
| PGM | Phosphoglucomutase |
| PK | Pyruvate Kinase |
| PMA | Plasma Membrane ATPase |
| PRO1 | gamma-glutamyl kinase |
| PRO2 | gamma-glutamyl phosphate reductase |
| RPE | Ribulose-Phosphate 3-Epimerase |
| RPI | Ribose 5-Phosphate Isomerase |
| SDH | Succinate Dehydrogenase |
| SUCD | Succinyl-CoA Synthetase |
| TAL | Transaldolase |
| TK | Transketolase |
| TPI | Triosephosphate Isomerase |
| ***Metabolites*** | |
| 2PG | 2-Phospho-D-glycerate |
| 34HPLAC* | 3,4-Dihydroxyphenylpyruvate (mapped to Phenylpyruvate measurements) |
| 34HPPYR* | 3,4-Dihydroxyphenyllactate (mapped to Phenyllactate measurements) |
| 3PG | 3-Phospho-D-glycerate |
| ACAC | Acetoacetate |

| | |
|---|---|
| ACCOA | Acetyl-CoA |
| ADP | Adenosine 5'-diphosphate |
| ALA | Alanine |
| ALAC | 2-Acetolactate |
| AMP | Adenosine 5'-monophosphate |
| ATP | Adenosine 5'-triphosphate |
| BPG | D-Glycerate 1,3-diphosphate |
| DHAP | Dihydroxyacetone phosphate |
| F6P | beta-D-Fructose 6-phosphate |
| FDP | beta-D-Fructose 1,6-bisphosphate |
| Ficyt c | Ferricytochrome c |
| Focyt c | Ferrocytochrome c |
| G1P | alpha-D-Glucose 1-phosphate |
| G3P | D-Glycerate 3-phosphate |
| G6P | alpha-D-Glucose 6-phosphate |
| GLC | Glucose |
| GLU | Glutamate |
| H20 | Water |
| H+ | Hydrogen |
| ILE | Isoleucine |
| LAC | Lactate |
| LEU | Leucin |
| LYS | Lysine |
| MET | Methionine |
| NADH | Nicotinamide adenine dinucleotide (reduced) |
| NAD+ | Nicotinamide adenine dinucleotide |
| O2 | Oxygen |
| ORN | Ornithine |
| PEP | Phosphoenolpyruvate |
| PHE | Phenylalanine |
| PLAC | Phenyllactate |
| PPYR | Phenylpyruvate |
| PRCOA | Propanoyl-CoA |
| PRPP | 5-Phosphoribosyl diphosphate |
| PUT* | Putrescine (mapped to N-acetylputrescine) |
| PYR | Pyruvate |
| St | Sulfate |
| SUCC | Succinate |
| THR | Threonine |
| TRP | Tryptophan |
| TYR | Tyrosine |
| VAL | Valine |

**Supplementary Table 4: Overview of screened deletion mutants for damage potential**

Damage phenotype of deletion mutants from the Noble collection (Noble *et al.* 2010 *Nat Genetics*) corresponding to differentially regulated genes during infection of *L. rhamnosus* colonized IECs. Mutant phenotype is classified as hypovirulent or hypervirulent when the damage caused to IECs was significantly lower or higher than the wild type, respectively, and non-significant (ns) when it was not.

| KO gene | Mutant phenotype | *P* value | KO gene | Mutant phenotype | *P* value |
|---|---|---|---|---|---|
| ace2 | Hypovirulent | 0,004 | orf19.5565 | ns | 0,9998 |
| ahr1 | Hypovirulent | <0,0001 | orf19.6874 | ns | 0,9807 |
| aox1 | ns | 0,9984 | orf19.7328 | Hypervirulent | 0,0838 |
| aro80 | ns | 0,3589 | orf19.7370 | ns | 0,9008 |
| asm3 | ns | 0,9993 | orf19.7516 | ns | 0,9996 |
| cfl2 | ns | 0,9993 | orf19.7554 | ns | 0,9832 |
| cht2 | ns | 0,9913 | pdk2 | Hypervirulent | 0,054 |
| cip1 | ns | 0,9818 | pex4 | ns | 0,9989 |
| cyb5 | ns | 0,9364 | pga13 | ns | 0,999 |
| fcr1 | ns | 0,5483 | pga45 | ns | 0,9912 |
| gal4 | ns | 0,9987 | pga7 | ns | 0,9842 |
| gis2 | ns | 0,9917 | pra1 | ns | 0,8833 |
| het1 | ns | 0,4653 | prk1 | ns | 0,9997 |
| hgt6 | ns | 0,9985 | prn2 | ns | 0,9999 |
| hyr1 | Hypervirulent | <0,0001 | prn4 | ns | 0,904 |
| kre5 | Hypovirulent | 0,0003 | ptp3 | Hypovirulent | <0,0001 |
| mal2 | ns | 0,9991 | pwp1 | ns | 0,3467 |
| mec3 | ns | 0,9996 | rbe1 | Hypervirulent | 0,0242 |
| mid1 | ns | >0,9999 | rbt4 | ns | 0,9817 |
| nik1 | ns | 0,9983 | rgs2 | Hypervirulent | 0,0511 |
| opt4 | ns | 0,9997 | rim13 | ns | 0,9985 |
| opt7 | Hypervirulent | 0,0572 | sef1 | ns | 0,9608 |
| orf19.1314 | ns | 0,8752 | snq2 | ns | 0,9981 |
| orf19.173 | ns | 0,999 | sod6 | ns | 0,9999 |
| orf19.215 | ns | 0,9995 | swi4 | ns | 0,9661 |
| orf19.3156 | ns | 0,9995 | tye7 | ns | 0,9997 |
| orf19.3395 | ns | >0,9999 | uga32 | ns | 0,9991 |
| orf19.3720 | ns | 0,9815 | vid27 | ns | 0,9994 |
| orf19.3982 | ns | >0,9999 | wor2 | ns | 0,9991 |
| orf19.4014 | ns | 0,9982 | ycp4 | ns | 0,6831 |
| orf19.4292 | Hypovirulent | <0,0001 | yhb1 | ns | 0,923 |
| orf19.4445 | ns | 0,9993 | ypt7 | Hypervirulent | 0,0766 |
| orf19.4459 | Hypervirulent | 0,0009 | ywp1 | ns | 0,9996 |
| orf19.449 | ns | 0,9993 | zcf27 | Hypervirulent | <0,0001 |
| orf19.4843 | ns | 0,624 | | | |
