# Supplementary material for: Lactobacillus rhamnosus colonisation antagonizes Candida albicans by forcing metabolic adaptations that compromise pathogenicity
Source: Nat Commun. 2022 Jun 9;13:3192. doi: 10.1038/s41467-022-30661-5 (PMC9184479; doi:10.1038/s41467-022-30661-5)
Supplement: Supplementary file 2 — Reporting Summary [file 41467_2022_30661_MOESM2_ESM.pdf]

nature portfolio

Corresponding author(s): Bernhard Hube

Last updated by author(s): Mar 29, 2022

Reporting Summary

Nature Portfolio wishes to improve the reproducibility of the work that we publish. This form provides structure for consistency and transparency in reporting. For further information on Nature Portfolio policies, see our Editorial Policies and the Editorial Policy Checklist.

Statistics

For all statistical analyses, confirm that the following items are present in the figure legend, table legend, main text, or Methods section.

n/a	Confirmed	
☐	☒	The exact sample size (*n*) for each experimental group/condition, given as a discrete number and unit of measurement
☐	☒	A statement on whether measurements were taken from distinct samples or whether the same sample was measured repeatedly
☐	☒	The statistical test(s) used AND whether they are one- or two-sided<br>*Only common tests should be described solely by name; describe more complex techniques in the Methods section.*
☒	☐	A description of all covariates tested
☐	☒	A description of any assumptions or corrections, such as tests of normality and adjustment for multiple comparisons
☐	☒	A full description of the statistical parameters including central tendency (e.g. means) or other basic estimates (e.g. regression coefficient) AND variation (e.g. standard deviation) or associated estimates of uncertainty (e.g. confidence intervals)
☐	☒	For null hypothesis testing, the test statistic (e.g. *F*, *t*, *r*) with confidence intervals, effect sizes, degrees of freedom and *P* value noted<br>*Give P values as exact values whenever suitable.*
☒	☐	For Bayesian analysis, information on the choice of priors and Markov chain Monte Carlo settings
☒	☐	For hierarchical and complex designs, identification of the appropriate level for tests and full reporting of outcomes
☒	☐	Estimates of effect sizes (e.g. Cohen's *d*, Pearson's *r*), indicating how they were calculated

Our web collection on statistics for biologists contains articles on many of the points above.

Software and code

Policy information about availability of computer code

Data collection	Growth curve and absorbance data was collected using Tecan i-control v 2.0.10.0. Microscopic images were acquired with Zeiss Zen3.1 (blue edition).
Data analysis	Data were analysed using Graphpad prism v 8, R v 1.2.5019 (packages: pHeatmap v1.0.12, dendextend v 1.15.2 , eulerr v6.1.0, ggbiplot v 0.55) Transcriptional data were analysed using Agilent GeneSpring v 14.9 and REVIGO. Metabolic data was analysed using MetaboAnalyst v 5 and GEM modeling was done using COBRApy in Python 3.6.4 and the IBM ILOG CPLEX Optimizer (v 12.8). Microscopic images were analysed with Zeiss Zen3.1 (blue edition).<br>The python code for the metabolic models and simulations is available at: https://github.com/mohammadmirhakkak/Calbicans_LRhamnosus_EpithelialCells_Interaction

For manuscripts utilizing custom algorithms or software that are central to the research but not yet described in published literature, software must be made available to editors and reviewers. We strongly encourage code deposition in a community repository (e.g. GitHub). See the Nature Portfolio guidelines for submitting code & software for further information.

Data

Policy information about availability of data

All manuscripts must include a data availability statement. This statement should provide the following information, where applicable:

- Accession codes, unique identifiers, or web links for publicly available datasets
- A description of any restrictions on data availability
- For clinical datasets or third party data, please ensure that the statement adheres to our policy

The authors declare that the data supporting the findings of this study are available within the paper and its supplementary information files. The source data are

provided as Source Data file. The transcriptomics data generated in this study has been deposited in the Array Express database under accession code: E-MTAB-11090: https://www.ebi.ac.uk/arrayexpress/experiments/E-MTAB-11090/
The untargeted metabolomics data generated in this study are provided in the Supplementary Data 1. The biomass objective function values and associated flux ranges for all reactions for all simulations and investigated media conditions generated in this study are provided in the Supplementary Data 2. The simulation sets over different fractions of required objective function values generated in this study are provided in the Supplementary Data 3. Databases used in this study include: Candida Genome Database (http://www.candidagenome.org/), RECON3d human metabolic model (www.vmh.life/files/reconstructions/Recon/3D.01/Recon3D_301.zip).

Field-specific reporting

Please select the one below that is the best fit for your research. If you are not sure, read the appropriate sections before making your selection.

[x] Life sciences [ ] Behavioural & social sciences [ ] Ecological, evolutionary & environmental sciences

For a reference copy of the document with all sections, see nature.com/documents/nr-reporting-summary-flat.pdf

Life sciences study design

All studies must disclose on these points even when the disclosure is negative.

Sample size	Sample size of each experiment is stated in Materials and methods, source data, Figure legends, and indicated in graphs shown as dot plot. No sample size calculation was performed, instead sample sizes were based on standards in the field.
Data exclusions	No data was excluded, unleas quality control controls warranted exclusion.
Replication	All experiments were independently repeated at least three times, unless otherwise stated. Details for each experiment are provided in Figure legends and Materials and methods.
Randomization	Randomization was not relevant to this study, all experiments were in-vitro. Fungi, bacteria and hostcells were cultured under identical conditions and unbiasedly allocated to well positions and treatments. Samples were harvested, processed and analyzed inrandom order when possible. Microscopic hyphal length measurements was performed by randomly chosen hyphae for quantification. Locations for represenative microscopy images were randomly selected using Zeiss Zen software Blue edition, and only modified when the image included well edges.
Blinding	Blinding was not relevant to this study, as most experiments were in-vitro and no subjective data analysis was performed. Microscopy pictures for hyphal length measurement were blinded to the researcher executing the measurements.

Reporting for specific materials, systems and methods

We require information from authors about some types of materials, experimental systems and methods used in many studies. Here, indicate whether each material, system or method listed is relevant to your study. If you are not sure if a list item applies to your research, read the appropriate section before selecting a response.

Materials & experimental systems

n/a	Involved in the study	
[x]	[ ]	Antibodies
[ ]	[x]	Eukaryotic cell lines
[x]	[ ]	Palaeontology and archaeology
[x]	[ ]	Animals and other organisms
[x]	[ ]	Human research participants
[x]	[ ]	Clinical data
[x]	[ ]	Dual use research of concern

Methods

n/a	Involved in the study	
[x]	[ ]	ChIP-seq
[x]	[ ]	Flow cytometry
[x]	[ ]	MRI-based neuroimaging

Eukaryotic cell lines

Policy information about cell lines

Cell line source(s)	Intestinal epithelial cells C2Bbe1 and HT29-MTX, obtained from ATCC, described in Methods section.
Authentication	Cell lines have been authenticated via commercial STR profiling (Eurofins Genomic).
Mycoplasma contamination	Mycoplasma contamination was routinely checked previously and the results were negative.
Commonly misidentified lines (See ICLAC register)	No misidentified cell lines used.
