# Supplementary material for: Lactobacillus rhamnosus colonisation antagonizes Candida albicans by forcing metabolic adaptations that compromise pathogenicity
Source: Nat Commun. 2022 Jun 9;13:3192. doi: 10.1038/s41467-022-30661-5 (PMC9184479; doi:10.1038/s41467-022-30661-5)
Supplement: Supplementary file 4 — Description of Additional Supplementary Files [file 41467_2022_30661_MOESM4_ESM.pdf]

Title: Supplementary Movie 1:
Description: Live microscopy of C. albicans filamentation in KBM supplemented with 50 mM cytosine. C. albicans was incubated at 37°C and 5% CO_2 for 20 h and brightfield pictures were taken every hour.

Title: Supplementary Movie 2:
Description: Live microscopy of C. albicans filamentation in KBM. C. albicans was incubated at 37°C and 5% CO_2 for 20 h and brightfield pictures were taken every hour.

Title: Supplementary Data 1:
Description: Metabolomics dataset. The relative concentrations of each metabolite in each condition are found as original data, scaled data and organised in pathways.

Title: Supplementary Data 2:
Description: Biomass objective function values and associated flux ranges for all reactions for all simulations and investigated media conditions generated in the study.

Title: Supplementary Data 3:
Description: Simulation sets over different fractions of required objective function values generated in this study.
